# Discordance between clinician- and patient-reported outcomes during radiotherapy for brain metastases: the prospective NeuroPRO study

**DOI:** 10.1016/j.ctro.2026.101283

**Published:** 2026-09-08

**Authors:** Cas Stefaan Dejonckheere, Yonah Lucas Layer, Anna-Laura Potthoff, Thomas Zeyen, Lara Caglayan, Franziska Grau, Shari Wiegreffe, Andrea Renate Glasmacher, Laura Kersting, Mario Richard Paco Kossmann, Markus Zeller, Younèss Nour, Katharina Layer, Christina Leitzen, Davide Scafa, Alexander Radbruch, Hartmut Vatter, Ambra Miriam Marx, Ulrich Herrlinger, Matthias Schneider, Gustavo Renato Sarria, Eleni Gkika, Julian Philipp Layer

**Affiliations:** a University of Bonn, University Hospital Bonn, Department of Radiation Oncology, 53127 Bonn, Germany; b University of Bonn, University Hospital Bonn, Department of Anesthesiology and Intensive Care Medicine, 53127 Bonn, Germany; c University of Bonn, University Hospital Bonn, Department of Neurosurgery, 53127 Bonn, Germany; d University of Bonn, University Hospital Bonn, Department of Neuro-Oncology, Center for Neurology and Integrated Oncology (CIO), 53127 Bonn, Germany; e University of Bonn, University Hospital Bonn, Department of Neuroradiology, 53127 Bonn, Germany; f University of Bonn, University Hospital Bonn, Department of Psychosomatic Medicine and Psychotherapy, 53127 Bonn, Germany; g University of Bonn, University Hospital Bonn, Institute of Experimental Oncology, 53127 Bonn, Germany

**Keywords:** Clinician-reported outcome, Patient-reported outcome, Radiotherapy, Whole-brain radiotherapy, Stereotactic radiosurgery, Brain metastasis, Quality of life

## Abstract

**Background:**

Clinician-reported outcomes (CROs) remain a central component of side effect assessment in radiotherapy, while patient-reported outcomes (PROs) are increasingly being integrated into standard practice to better capture the subjective symptom burden experienced by patients. Agreement between CRO and PRO has not been prospectively studied in patients undergoing radiotherapy for brain metastases.

**Methods:**

NeuroPRO was a prospective longitudinal cohort study enrolling patients receiving radiotherapy for brain metastases. CROs were assessed using predefined CTCAE (version 5.0) items, while PROs were collected using the EORTC QLQ-C30 and BN20 at baseline, end of treatment, and first follow-up. Upon completion of radiotherapy, decision regret was evaluated additionally using the Ottawa Decision Regret Scale. Chance-corrected agreement between CRO and PRO was quantified for each item using Gwet's second-order agreement coefficient (AC2). Longitudinal differences were additionally explored using two-way ANOVA and agreement was further characterized using Bland-Altman analyses with calculation of limits of agreement (LoA).

**Results:**

Sixty-three patients were included, completing 278 questionnaires and yielding 4448 matched CRO-PRO item comparisons. Across 27 of 32 shared symptoms (84%), PRO scores were higher than CRO scores, indicating *systematic* symptom underestimation by clinicians. Chance-corrected agreement (Gwet's AC2) was fair to moderate for psychological and neurological symptoms (worry AC2 = 0.30; depression AC2 = 0.39; drowsiness AC2 = 0.44) and substantial to almost perfect for gastrointestinal and treatment-related symptoms (vomiting AC2 = 0.96; seizures AC2 = 0.94; nausea AC2 = 0.92), corroborating the pattern observed with mean-based comparisons. The largest single-item discrepancies were observed for dyspnea (bias 1.00; 95% LoA 0.85–1.14; group *p* < 0.001), insomnia (bias 0.76; 95% LoA 0.22–1.29; group *p* < 0.001), and depression (bias 0.75; 95% LoA 0.39–1.12; group *p* < 0.001). Symptom clustering demonstrated higher agreement for directly treatment-related effects and physical functioning, whereas agreement was substantially lower for emotional and cognitive domains. Patient-reported global health status remained stable after radiotherapy, and decision regret was low.

**Conclusion:**

Sole clinician assessment systematically underestimates symptom burden in patients undergoing radiotherapy for brain metastases, particularly within psychological domains and somatic symptoms not readily attributable to radiotherapy. These findings reveal a structural perception gap and support the routine integration of PROs into interdisciplinary neuro-oncologic care to enhance symptom recognition and promote patient-centered management (DRKS00031480).

## Highlights

• Prospective NeuroPRO study compared CROs and PROs during RT for brain metastases.
• Clinicians underestimated symptom burden in 84% of matched items.
• Greatest discordance involved dyspnea, insomnia, and depression.
• Agreement was higher for RT-related than emotional and cognitive symptoms.
• Findings support routine PRO integration in neuro-oncologic care.

## 1 Introduction

Radiotherapy (RT) plays a central role in the treatment of cancer with more than half of all oncologic patients receiving irradiation [1]. In the management of brain metastases (BMs) in particular, RT remains essential to achieve sustained local tumor control, either in a definitive or (neo)adjuvant setting [2], [3], [4], [5], [6]. Historically, whole-brain radiotherapy (WBRT) represented the standard approach [2]. More recently, stereotactic radiosurgery (SRS) has become an effective modality for patients with a limited number of BMs, reducing the risk of treatment-related neurocognitive impairment without compromising survival [3], [4], [7], [8].

Treatment tolerability and the impact on quality of life (QoL) are critical considerations in cranial RT. Traditionally, RT-associated side effects have been assessed using clinician-reported outcomes (CROs), enabling standardized side effect grading and guiding supportive care. However, CROs alone may incompletely capture the subjective symptom burden experienced by patients. Patient-reported outcomes (PROs) have therefore gained increasing attention as a complementary measure that more directly reflects patient experience and functional well-being [9], [10].

In several extracranial malignancies, including breast, prostate, anal, rectal, and cervical cancer, comparative studies of CROs and PROs have demonstrated systematic discrepancies, with clinicians frequently underestimating both symptom incidence and severity [11], [12], [13], [14], [15]. Such divergence raises concerns that exclusive reliance on CROs may result in underrecognition and thus undertreatment of clinically meaningful side effects and clinical conditions. Despite the distinct neurocognitive and neuropsychiatric vulnerability associated with cranial irradiation, prospective data evaluating concordance between CROs and PROs in patients receiving RT for BMs are lacking. Here, we present results from the prospective observational NeuroPRO study, which longitudinally assessed discordance between CROs and PROs in patients undergoing cranial RT.

## 2 Materials and methods

### 2.1 Trial design and oversight

**Fig. 1:**
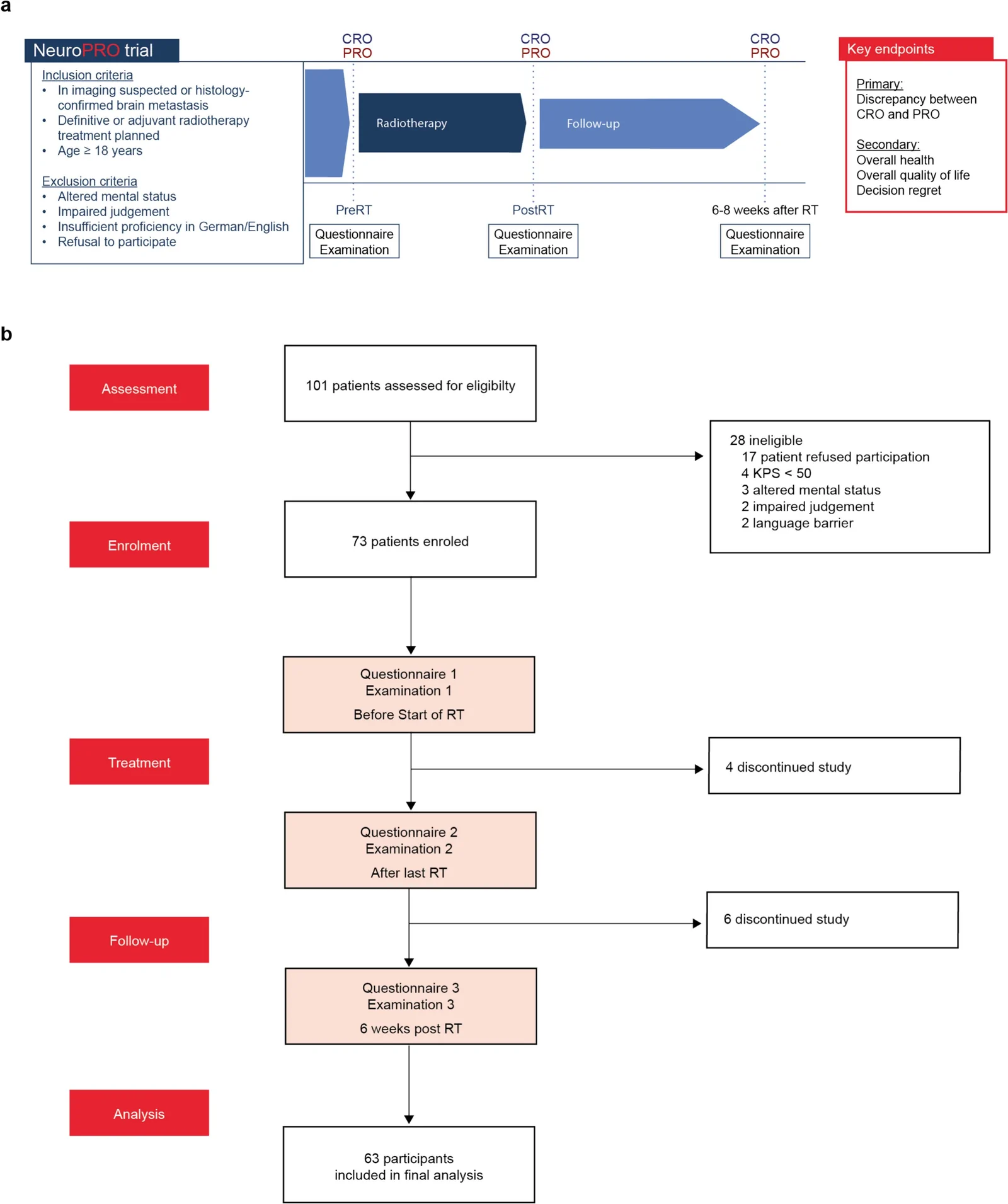
(a) Summary of the NeuroPRO study design. (b) Flowchart of patient inclusion. CRO, clinician-reported outcome; PRO, patient-reported outcome; RT, radiotherapy; KPS; Karnofsky performance score.

NeuroPRO was a prospective observational trial, which longitudinally evaluated discordance between CROs and PROs in patients undergoing cranial RT at our tertiary neuro-oncologic referral center (Fig. 1a). Herein, we report the final results for patients with BMs undergoing SRS, fractionated stereotactic RT, or WBRT. The primary endpoint was the overall discrepancy between CROs and PROs (single items and predefined clusters) including changes of agreement over time. Secondary endpoints included overall health and QoL across the collection timepoints as well as decision regret after completion of RT.

From January 2023 to June 2024, all patients scheduled for RT of at least one BM at our comprehensive academic cancer center were prospectively screened for inclusion. Eligibility criteria comprised age ≥ 18 years and a Karnofsky Performance Score (KPS) ≥ 50, and imaging suggestive of or histology confirming BM of a solid tumor. Exclusion criteria were altered mental status, impaired judgment, and insufficient understanding of the German or English language. Altered mental status and impaired judgment were clinically assessed at screening and were considered exclusion criteria only when they interfered with the patient's ability to provide informed consent, understand the study procedures, or reliably complete the study assessments. No formal cognitive screening threshold was applied.

This prospective study was approved by the local Institutional Review Board (456-EP/22) and conducted in accordance with the Declaration of Helsinki. Written informed consent was obtained from all participants prior to participation. Trial participants did not receive financial compensation. The trial was registered in the German Clinical Trials Registry (DRKS00031480) and has completed accrual.

### 2.2 Radiation treatment

All patients were treated according to national and international guidelines following case discussion and consensus in a multidisciplinary tumor board. RT was delivered either in a definitive or an adjuvant setting.

For immobilization, an individual thermoplastic fixation mask was tailored for each patient. Treatment planning included a planning computed tomography (CT) scan and a rigidly co-registered contrast-enhanced magnetic resonance imaging (MRI), obtained within seven days prior to treatment. Target volume delineation followed international consensus guidelines. The margin from gross tumor volume (GTV) to planning target volume (PTV) was 3–5 mm for WBRT and 1 mm for stereotactic RT according to institutional standards.

Dose prescription followed International Commission on Radiation Units and Measurements (ICRU) recommendations, with dose limits of 95–107% for WBRT and 99–120% for SRS. All treatments were delivered on a TrueBeam STx linear accelerator (Varian Medical Systems, Siemens Healthineers, Palo Alto, CA, USA) using 6–10 MV photon beams. Daily image guidance was performed using on-board cone-beam CT for WBRT and ExacTrac stereoscopic positioning (Brainlab, Munich, Germany) for stereotactic RT.

### 2.3 Clinician-reported outcome

CRO was assessed at baseline (prior to the first fraction), at completion of RT, and at the first follow-up visit approximately six weeks after RT completion. In patients undergoing single-fraction SRS, two separate assessments were performed on the treatment day: the baseline assessment immediately before SRS and the end-of-treatment assessment after completion of irradiation. Side effects were graded according to the National Cancer Institute (NCI) Common Terminology Criteria for Adverse Events (CTCAE), version 5.0 (***Supplement A***) [16]. Assessments were performed by the treating radiation oncologist based on structured patient interviews and standardized neurological examination. All predefined items were evaluated at each timepoint, including those scored as grade 0 (ie, absence of a sign or symptom).

### 2.4 Patient-reported outcome

PRO was assessed using the European Organisation for Research and Treatment of Cancer (EORTC) Quality of Life Questionnaire (QLQ) Core 30 (C30) and the brain-specific module (BN20). At the first follow-up after RT completion, the Ottawa Decision Regret Scale was completed additionally. All questionnaires were paper-based and completed by patients at the same predefined timepoints as clinician assessments (***Supplement B***) [17]. Scoring was performed using each questionnaire's respective manual.

### 2.5 Matching of clinician- and patient-reported items

Individual CTCAE items were matched to QLQ-C30 or BN20 items when both instruments assessed the same symptom or a closely corresponding functional domain. Matching was performed at the discretion of the investigators, on conceptual and clinical grounds. All pairings were defined before study initiation including conduct of the agreement analysis. A complete overview of item pairs is provided in ***Supplement C***.

### 2.6 Statistical analysis

Patients were included in the analysis if at least one clinician- and patient-reported assessment was available for the same symptom at the same timepoint. Descriptive statistics, including mean, median, standard deviation (SD), and range were calculated for all applicable clinical variables.

To formally quantify agreement between CROs and PROs for each of the 32 predefined item pairs, Gwet's second-order chance-corrected agreement coefficient (AC2) with quadratic weights was calculated. For domain scores comprising multiple EORTC subitems (eg, fatigue, limitations in activities of daily living, pain), the resulting non-integer mean score was rounded to the nearest whole CTCAE grade beforehand. AC2 was calculated both pooled across all timepoints (primary analysis) and stratified by timepoint (baseline, end of treatment, first follow-up) to assess whether the degree of agreement changed over the course of treatment. AC2 ranges from 0 (agreement no better than chance) to 1 (perfect agreement); resulting values were categorized descriptively using the Landis & Koch benchmark scale (poor ≤0, slight 0.01–0.20, fair 0.21–0.40, moderate 0.41–0.60, substantial 0.61–0.80, almost perfect 0.81–1.00) for descriptive orientation only.

As a secondary exploratory analysis, differences between CROs and PROs across timepoints were analyzed using two-way analysis of variance (ANOVA) with reporting method (CRO, PRO) and timepoint (baseline, end of treatment, follow-up) specified as fixed factors. Clinically related symptoms were grouped into predefined clusters to identify domains with particularly pronounced discrepancies between clinician- and patient-reported assessments. Mixed effects modelling was considered but not applied due to incomplete repeated measures across timepoints and limited sample size per symptom domain, which could lead to model instability. Missing values were not imputed. For each item, two-way ANOVA tested the main effect of reporting method (ie, the overall difference in severity score between CRO and PRO, irrespective of timepoint; referred to as the “group effect”), the main effect of timepoint (ie, whether severity changed over the course of treatment, irrespective of reporting method; the “time effect”), and the reporting method × timepoint interaction (ie, whether the magnitude or direction of CRO-PRO discordance itself changed over time). The dependent variable was the item-level severity score (CTCAE grade or corresponding EORTC item/domain score); reporting method and timepoint were the independent fixed factors. Tukey's multiple comparisons test was applied for post-hoc pairwise comparisons between timepoints.

Agreement between CROs and PROs was further explored using Bland-Altman analyses to estimate systematic bias and corresponding 95% limits of agreement (LoA) for individual symptom domains. Systematic bias was defined as the mean difference between PRO and CRO scores. Wider LoA were interpreted as indicating greater variability and reduced interchangeability between reporting methods. A clinically relevant lack of agreement was pragmatically defined based on clinical interpretability as a mean difference exceeding one severity grade (in line with methodology of similar trials for other entities).

All statistical tests were two-sided, with statistical significance defined as *p* < 0.05. When appropriate, 95% confidence intervals (CI) are provided. Statistical analyses were performed with *R* version 4.5.2 (*R* Foundation for Statistical Computing, Vienna, Austria) and GraphPad Prism version 10 (GraphPad Software, San Diego, CA, USA). Figures and graphs were created using *R* (*tidyverse*, *irrCAC*, and *ggradar* packages), GraphPad Prism, and Adobe Illustrator 2023 (Adobe Inc., Mountain View, CA, USA).

## 3 Results

### 3.1 Trial design, patient and treatment characteristics

Between January 2023 and June 2024, 63 patients were included in the final analysis (Fig. 1b), of whom 48% were female. The median age was 63 years (range, 44–87). Nineteen percent of patients underwent WBRT, whereas 81% received stereotactic RT for a median of 1 lesion (range, 1–10). The most common primary tumor was lung cancer (43%), followed by melanoma (27%) and breast cancer (10%). Forty percent of patients had received prior cranial RT. Thirty-eight percent of patients received adjuvant RT after surgical resection. Systemic disease was limited (ie, oligometastatic or oligoprogressive, defined as 1–5 active metastases at the time of radiotherapy) in 24% and overt in 51% of patients. Fifty-six percent of patients received any form of systemic therapy in parallel. An overview of patient and treatment characteristics is provided in Table 1.

**Table 1:** Summary of patient and treatment characteristics (*n* = 63). KPS, Karnofsky Performance Score; DS-GPA, disease-specific Graded Prognostic Assessment; RT, radiotherapy; SRS, stereotactic radiosurgery; WBRT, whole-brain radiotherapy.

	*n* (%)
Median age (range), years	63 (44–87)
Sex	
Male	33 (52)
Female	30 (48)
Ethnicity	
Caucasian	61 (97)
Other	2 (3)
KPS, %	
100	6 (10)
80–90	38 (60)
60–70	17 (27)
50	2 (3)
Median DS-GPA (range)	2 (0–4)
Primary tumor	
Lung	27 (43)
Melanoma	17 (27)
Breast	6 (10)
Other	13 (21)
History of prior cranial RT	25 (40)
History of prior cranial surgery	24 (38)
RT modality	
Single SRS	33 (52)
Multiple SRS	18 (29)
WBRT	12 (19)
Median number of lesions ⁎ (range), *n*	1 (1−10)
Systemic disease at the time of RT	
Overt	32 (51)
Limited ⁎⁎	15 (24)
No	16 (25)
Concomitant therapy	
Immunotherapy	22 (35)
Chemotherapy	13 (21)
None	28 (44)
Corticosteroids intake at baseline	15 (24)

⁎ In non-WBRT patients.

⁎⁎ Oligometastatic or oligoprogressive disease, defined as 1–5 active metastases at the time of radiotherapy.

### 3.2 Agreement between clinician- and patient-reported outcomes

**Fig. 2:**
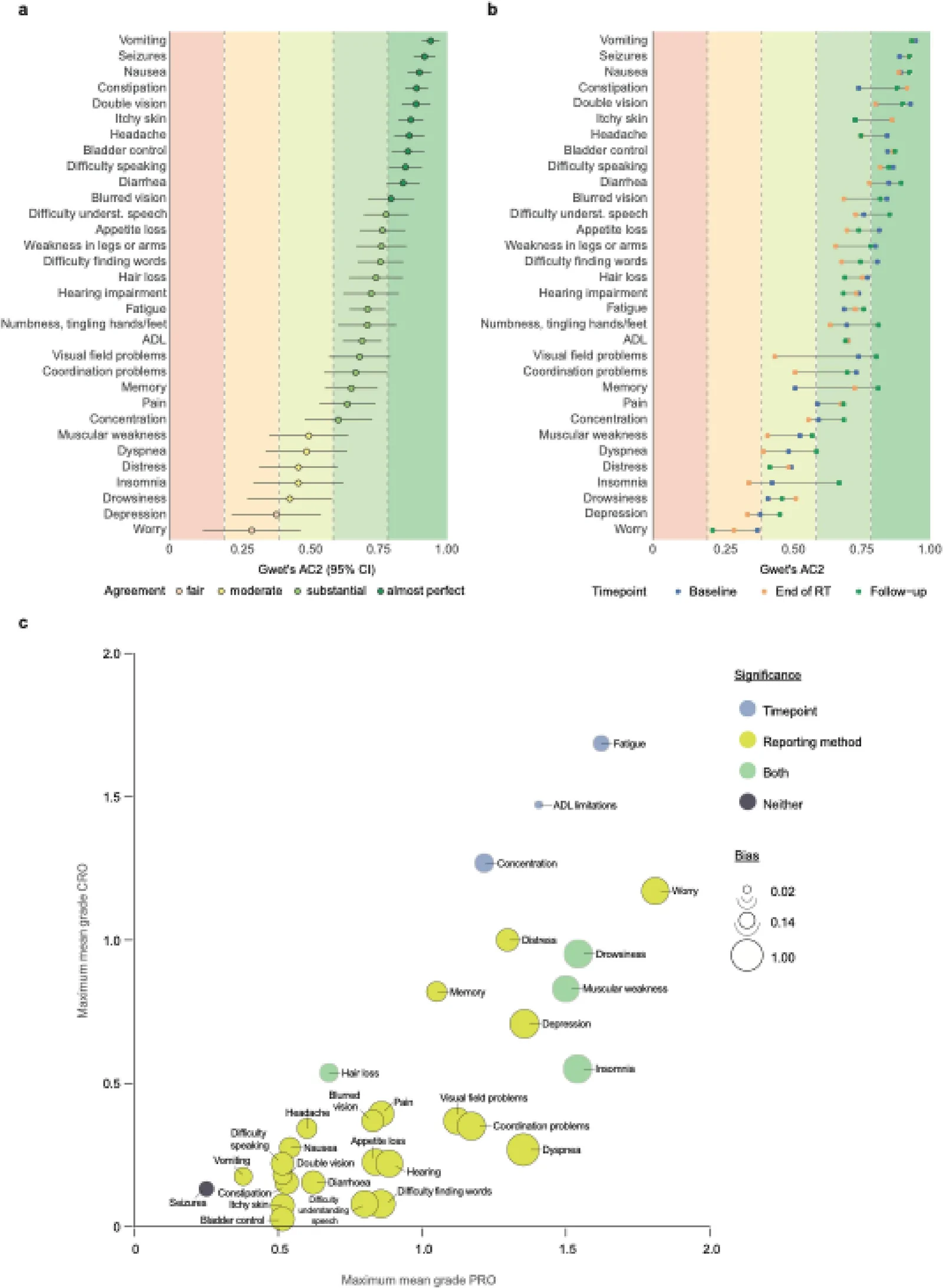
(a) Forest plot showing chance-corrected agreement (Gwet's AC2; quadratic weights) between CRO and PRO for 32 individual items, pooled across all timepoints. Points show the AC2 estimate with 95% confidence intervals; background shading indicates Landis & Koch benchmark categories (poor/slight, fair, moderate, substantial, almost perfect), provided for descriptive orientation only. Items are sorted by increasing AC2. (b) AC2 for the same 32 items, stratified by timepoint (baseline, end of treatment, first follow-up); items are shown in the same order as in (a). (c) Bubble plot illustrating the maximum mean grading per symptom item for CRO (*y-axis*) and PRO (*x-axis*). Bubble colour indicates statistical significance from two-way ANOVA (*p* < 0.05) for timepoint (*blue*), reporting method (*yellow*), both factors (*green*), or neither (*gray*). Bubble sizes reflect the magnitude of Bland-Altman bias as defined in the legend. Underst., understanding; ADL, activities of daily living; AC2, Gwet's second-order agreement coefficient; CI, confidence interval; RT, radiotherapy; CRO, clinician-reported outcome; PRO, patient-reported outcome. (For interpretation of the references to colour in this figure legend, the reader is referred to the web version of this article.)

Overall, 278 questionnaires were completed and compared, resulting in 4448 CRO-PRO item pairs. The direction of discrepancy was highly consistent. Overall, in 27 of 32 items (84%), patients reported higher symptom intensity than clinicians (***Supplement D***). Chance-corrected agreement (Gwet's AC2), pooled across timepoints, is shown for all items in Fig. 2a and ***Supplement E***. Highest agreement was observed for vomiting (AC2 = 0.96; 95% CI 0.93–0.99), seizures (AC2 = 0.94; 95% CI 0.90–0.97), and nausea (AC2 = 0.92; 95% CI 0.87–0.96), whereas the lowest agreement occurred for worry (AC2 = 0.30; 95% CI 0.12–0.48), depression (AC2 = 0.39; 95% CI 0.23–0.56), and drowsiness (AC2 = 0.44; 95% CI 0.28–0.60). When stratified by timepoint (Fig. 2b; ***Supplement F***), the degree of agreement remained broadly stable for most items; however, several items (including visual field problems, insomnia, memory, and coordination problems) showed a spread in AC2 exceeding 0.20 across timepoints (***Supplement G***).

Two-way ANOVA demonstrated a significant main effect of reporting method (CRO versus PRO; group *p* < 0.05) for most symptoms, while interaction effects were non-significant, suggesting that the observed discrepancies remained stable over time (***Supplement H***).

With a mean CTCAE grading of 1.7 at completion of RT, fatigue was the item with the highest grading in both CRO and PRO, showing a significant time effect (*p* < 0.001) without significant group effect (*p* = 0.187), indicating temporal variation in symptom severity but comparable ratings between clinicians and patients over time. In contrast, depression demonstrated a strong group effect (*p* < 0.001), with PRO exceeding CRO scores across all timepoints (mean difference 0.75; 95% CI 0.54 to 0.97), but no significant interaction (*p* = 0.425), implying that patients reported more depression than clinicians across all timepoints. A similar pattern was observed, among other, for dyspnea (group effect *p* < 0.001; mean difference 1.00; 95% CI 0.78 to 1.21), insomnia (group effect *p* < 0.001; mean difference 0.76; 95% CI 0.53 to 0.98), and anorexia (group effect *p* < 0.001; mean difference 0.59; 95% CI 0.41 to 0.76). Nausea and emesis showed a smaller but still significant discrepancy (*p* = 0.001; mean difference 0.29; 95% CI 0.11 to 0.49 for nausea and *p* = 0.009; mean difference 0.18; 95% CI 0.05 to 0.32 for emesis). Notably, several neurologic symptoms also demonstrated pronounced discrepancies between CRO and PRO: drowsiness (*p* < 0.001; mean difference 0.75; 95% CI 0.54 to 0.96), coordination problems (*p* < 0.001; mean difference 0.70; 95% CI 0.50 to 0.91), and muscular weakness (*p* < 0.001; mean difference 0.65; 95% CI 0.41 to 0.88) showed consistently higher patient-reported scores compared to clinician assessments. In contrast, some functional outcomes showed stronger agreement between both perspectives; for example, limitations in activities of daily living did not demonstrate a significant group effect (*p* = 0.833; mean difference 0.02; 95% CI –0.19 to 0.24) (***Supplement H***).

Bland-Altman analysis confirmed *systematic* positive bias favoring higher PRO scores. The largest bias was observed for dyspnea (bias 1.00; 95% LoA 0.85 to 1.14), followed by insomnia (bias 0.76; 95% LoA 0.22 to 1.29), and depression (bias 0.75; 95% LoA 0.39 to 1.12). Fatigue demonstrated a comparably small bias (bias 0.13; 95% LoA −0.20 to 0.47), suggesting overall closer agreement between assessment methods (Fig. 2c).

**Fig. 3:**
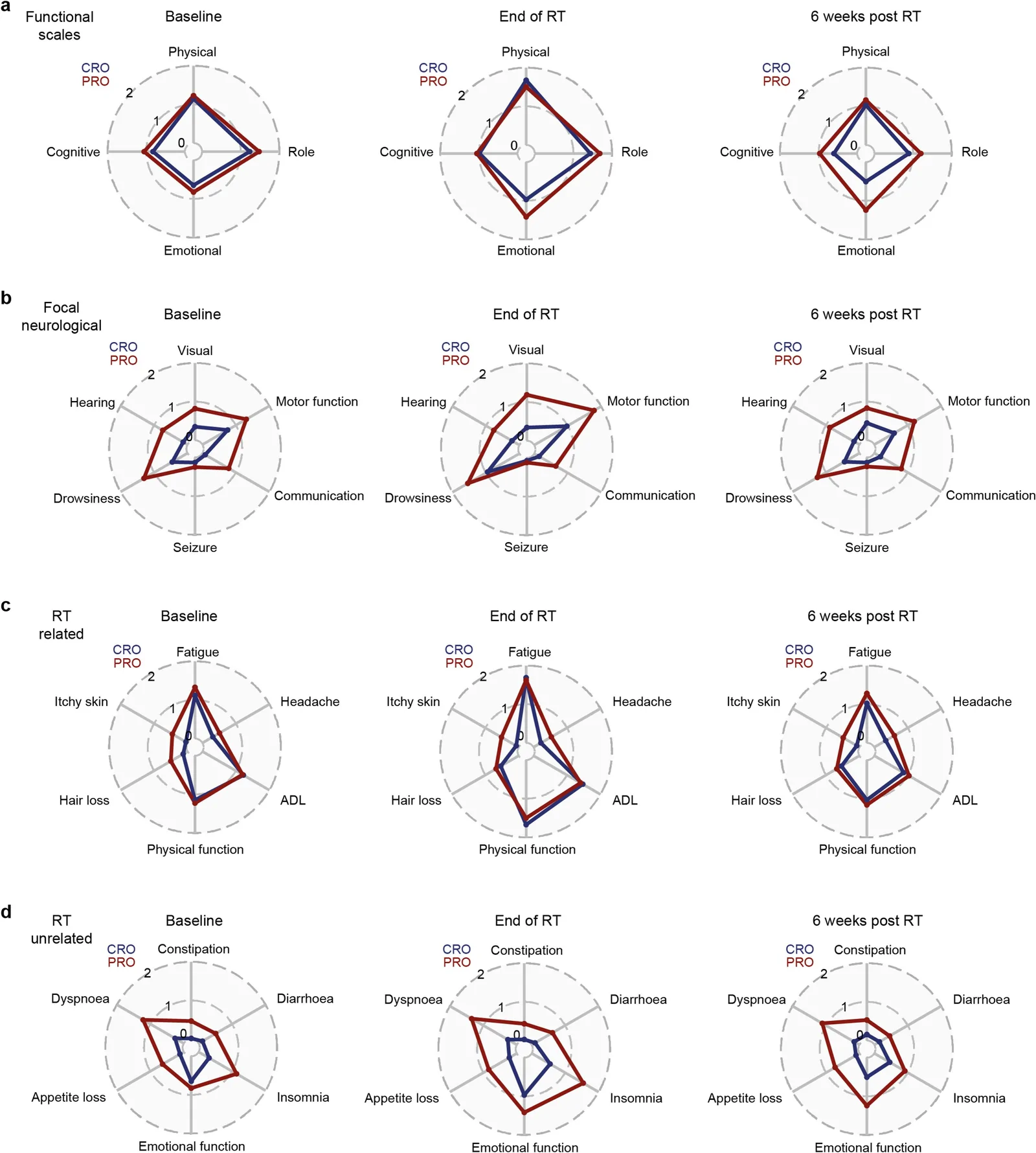
Radar charts showing agreement between CRO (*blue*) and PRO (*red*) for predefined clusters: (a) functional scales, (b) focal neurological, (c) RT-related, and (d) RT-unrelated. CRO, clinician-reported outcome; PRO, patient-reported outcome; RT, radiotherapy. (For interpretation of the references to colour in this figure legend, the reader is referred to the web version of this article.)

When symptoms were grouped into predefined clinical clusters, domain-specific differences in agreement became more apparent. Among functional scales, agreement was highest for physical functioning and progressively lower for cognitive, role, and emotional functioning (Fig. 3a). A similar pattern emerged across focal neurological domains, where agreement varied substantially depending on the type of deficit (Fig. 3b). Notably, symptoms within the RT-related cluster demonstrated better overall agreement compared with RT-unrelated symptoms, indicating that clinician ratings aligned more closely with patient reports for treatment-associated effects than for broader symptom burden (Fig. 3c, d).

### 3.3 Patient-reported overall health and quality of life

**Fig. 4:**
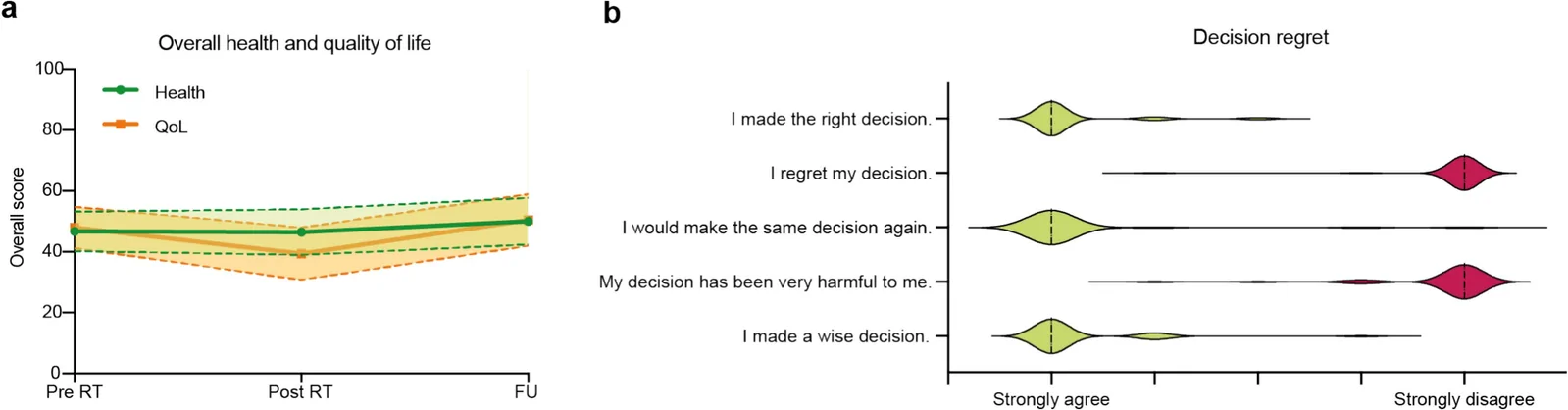
(a) Overall self-reported health and quality of life of participants. (b) Assessment of decision regret regarding radiotherapy at the first follow-up visit. QoL, quality of life; RT, radiotherapy; FU, follow-up.

Self-reported overall health remained stable over time, whereas self-reported QoL showed a transient decline at the end of treatment, followed by recovery at follow-up, consistent with acute treatment-related effects (Fig. 4a).

### 3.4 Decision regret

Overall, decision regret scores were low, with a mean of 5.3 ± 11.8 at the time of first follow-up (Fig. 4b). The majority of patients reported no or only mild regret. Moderate-to-high regret (> 25) was observed in 6.1% of cases only.

## 4 Discussion

To our knowledge, this study represents the first prospective longitudinal evaluation of agreement between CRO and PRO in patients undergoing cranial radiotherapy for brain metastases. The NeuroPRO study reveals a consistent and clinically meaningful discordance between CRO- and PRO-based symptom assessment. This finding is of particular relevance in a population characterized by neurocognitive and neuropsychiatric vulnerability and often limited life expectancy, for which preservation of quality of life may frequently be of equal or even greater importance than marginal survival gains.

Across the majority of assessed symptoms, clinicians systematically underestimated patient-reported burden. This discrepancy was particularly pronounced for less externally observable symptoms, particularly psychological domains such as depressive symptoms or insomnia, whereas agreement was comparatively higher for physical functioning and RT-related side effects, which are routinely evaluated during on-treatment visits. Importantly, the magnitude and direction of disagreement remained stable across timepoints, suggesting a structural perception gap inherent to clinician-based assessments rather than a transient phenomenon tied to a specific treatment phase.

These findings align with a growing body of evidence across different malignancies treated with RT, showing limited concordance between CROs and PROs (Table 2). Predominantly retrospective studies in breast, prostate, anal, rectal, and cervical cancer have previously demonstrated that clinicians underreport both frequency and severity of acute radiotherapy-related symptoms in comparison with affected patients. Behroozian et al. observed systematic underestimation of symptoms associated with radiation dermatitis in 777 breast cancer patients, with low-to-moderate concordance across most items and timepoints [11]. Similarly, Rammant et al., Tom et al., Flores et al., and Kirchheiner et al. reported poor agreement for acute gastrointestinal and genitourinary side effects during pelvic RT, with clinicians consistently rating symptom severity lower than patients [12], [13], [14], [15]. Importantly, these prior investigations largely focused on observable somatic symptoms. Our results extend this literature by suggesting that discordance may be even more pronounced for affective symptom domains, particularly emotional functioning, where clinician assessment appears least aligned with patient experience.

**Table 2:** Selected literature comparing CRO and PRO for the assessment of acute side effects across different tumor entities treated with curative radiotherapy.

Author, year	*n*	Entity	Outcome
Behroozian et al., 2021	777	breast cancer	all items significantly underreported by clinicians, low-to-moderate level of concordance
Rammant et al., 2018	160	prostate cancer	low agreement for GI and GU side effects
Tom et al., 2017	78	anal cancer	discrepancies between CRO and PRO
Flores et al., 2012	197	rectal cancer	discrepancies between CRO and PRO
Kirchheiner et al., 2012	223	cervical cancer	mismatch in reporting of symptoms, high risk of underestimation by clinicians

While concordance gaps are well documented, the reasons underlying these observations remain incompletely understood. A large analysis by Jagsi et al. further quantified the magnitude of this phenomenon. Among nearly 10,000 patients, clinicians failed to recognize at least one substantial (moderate-to-severe) symptom in approximately half of cases [18]. Underrecognition was more frequent in younger patients and in ethnic minority populations, underscoring that discordance is not random but influenced by patient- and provider-level factors.

A relevant proportion of patients in this study (40%) previously underwent cranial RT. These patients may have potentially perceived and reported symptoms differently because of previous treatment experience, cumulative toxicities, or greater awareness of treatment-related effects, whereas clinicians inevitably tend to assess toxicity primarily in the context of the current treatment course and may thus underestimate the effects of previous treatment lines. Consequently, the degree of CRO-PRO discordance may differ between reirradiated patients and those undergoing cranial RT for the first time. Due to the limited subgroup size in this cohort, this is an aspect that remains to be elucidated in a future larger study or pooled analysis.

Overall, the observed gap between CRO and PRO is likely multifactorial. First, clinicians and patients might evaluate fundamentally different constructs. Clinician scoring systems such as CTCAE prioritize observable signs and predefined toxicity categories, whereas patients report subjective lived experiences. Symptoms such as mood disturbance, sleep disruption, or dyspnea are inherently internal and therefore more susceptible to underrecognition. Second, repeated exposure to (severe) side effects may create a normalization or habituation effect among clinicians, as discordance has been shown to increase as treatment progresses [19]. What represents a substantial burden to a patient may be perceived as routine in a high-acuity oncology environment. Third, patients themselves may undercommunicate symptoms due to time pressure, fear of treatment interruption, or concern that reporting symptoms could jeopardize a potentially life-prolonging therapy. This dynamic may be amplified in patients with BMs, who often face existential decision-making and might prioritize continuation of treatment. Fourth, structural healthcare factors, including time constraints in busy clinical departments and lack of standardized PRO integration, may limit opportunities for nuanced symptom exploration. The fact that agreement was better for RT-related than for RT-unrelated symptom burden suggests that clinicians are most accurate when symptoms fit expected treatment frameworks. Broader, holistic symptom domains (emotional distress, cognitive functioning, global well-being) require both a more interdisciplinary and individualized, patient-centered approach.

Reliance on CRO alone thus risks undertreatment of clinically meaningful symptoms. The adoption of PROs is therefore not simply methodological refinement but a necessary evolution toward patient-centered oncology and of particular importance in the field of neuro-oncology [20]. However, integration must go beyond parallel measurement. Because PRO items cannot be directly mapped onto CRO scales without conceptual distortion, new hybrid instruments may be required to bridge the interpretive gap. In practical terms, this argues for routine PRO screening during cranial RT visits, particularly for psychological and affective symptoms that are otherwise easily missed.

Several limitations warrant consideration. First, the study cohort was heterogeneous with respect to systemic therapy, with approximately half of patients receiving any concomitant systemic treatment. While this may reflect real-world practice, potential confounding effects cannot be excluded. Second, because participation required sufficient cognitive capacity to provide informed consent and complete questionnaires, patients with severe cognitive impairment may be underrepresented, potentially limiting generalizability to the most neurologically impaired subgroup of patients with BMs. Third, follow-up duration was relatively short. Longer observation might reveal delayed neurocognitive sequelae or evolving decision regret, particularly among patients undergoing WBRT. However, extended longitudinal assessment in this population is intrinsically limited by attrition due to disease progression and mortality, as recently illustrated in another clinical trial [21]. The loss to follow-up observed in our cohort reflects this structural challenge and may have also introduced survivorship bias, potentially attenuating symptom burden at later timepoints. Fourth, although longitudinal differences were analyzed using two-way ANOVA, results of the repeated measures structure should not be overstated, as no model using higher-parameter mixed effects approaches was employed. While mixed models may offer statistical advantages, their application in a comparatively small cohort with incomplete follow-up increases the risk of unstable estimates. Consequently, these findings should primarily be viewed as hypothesis-generating. Finally, comparison between CRO and PRO instruments necessitates mapping across two inherently different measurement frameworks. These capture related but non-identical constructs and cannot be translated without conceptual loss. This limitation is shared by all CRO-PRO comparison studies as integrated cross-validated instruments showing psychometric equivalence are not yet established. The ordinal harmonization used in the present study therefore only preserves the relative ordering of symptom severity. Nevertheless, we employed the most widely used and internationally accepted instruments to best reflect real-world clinical practice and maximize clinical relevance. The consistent predominance of discordant pairs in which patients reported a higher severity category indicates that clinician assessment alone may fail to capture part of the subjective symptom burden [22].

Given the absence of prior prospective data in cranial RT, independent validation in larger multicenter cohorts and comparison with primary brain tumor populations are warranted. The observed discordance between CRO and PRO strongly supports the integration of PRO endpoints into future RT trials, particularly in settings where quality of life is a central therapeutic objective. Incorporating PROs into routine clinical practice may further enhance symptom detection and facilitate timely, patient-centered oncologic care.

## 5 Conclusion

In patients undergoing cranial radiotherapy for brain metastases, clinician assessment alone does not fully capture the lived symptom burden. Systematic underestimation, especially for psychological symptoms, highlights the necessity of integrating patient-reported outcomes into clinical trials as well as routine care. Improved symptom recognition is not merely descriptive, but contributes to more comprehensive, patient-centered oncologic care.

## CRediT authorship contribution statement

**Cas Stefaan Dejonckheere:** Writing – review & editing, Writing – original draft, Visualization, Validation, Supervision, Project administration, Methodology, Investigation, Formal analysis, Data curation, Conceptualization. **Yonah Lucas Layer:** Writing – review & editing, Formal analysis. **Anna-Laura Potthoff:** Writing – review & editing. **Thomas Zeyen:** Writing – review & editing. **Lara Caglayan:** Writing – review & editing. **Franziska Grau:** Writing – review & editing. **Shari Wiegreffe:** Writing – review & editing. **Andrea Renate Glasmacher:** Writing – review & editing. **Laura Kersting:** Writing – review & editing. **Mario Richard Paco Kossmann:** Writing – review & editing. **Markus Zeller:** Writing – review & editing. **Younèss Nour:** Writing – review & editing. **Katharina Layer:** Writing – review & editing. **Christina Leitzen:** Writing – review & editing. **Davide Scafa:** Writing – review & editing. **Alexander Radbruch:** Writing – review & editing. **Hartmut Vatter:** Writing – review & editing. **Ambra Miriam Marx:** Writing – review & editing. **Ulrich Herrlinger:** Writing – review & editing. **Matthias Schneider:** Writing – review & editing. **Gustavo Renato Sarria:** Writing – review & editing. **Eleni Gkika:** Writing – review & editing. **Julian Philipp Layer:** Writing – review & editing, Visualization, Validation, Supervision, Methodology, Investigation, Formal analysis, Data curation.

## Funding information

This research did not receive any external funding.

## Declaration of competing interest

CSD reports honoraria from Carl Zeiss Meditec and Siemens Healthineers outside the submitted work. UH reports advisory and/or speaker fees from OncoMAGNETx, Medac, and Servier. GRS reports research grants from Carl Zeiss Meditec; personal/consulting or advisory fees from AstraZeneca, Carl Zeiss Meditec, Guerbet, Medwave Clinical Trials, and Roche Pharma; and travel expenses from AstraZeneca, Carl Zeiss Meditec, Guerbet, and Medwave Clinical Trials outside the submitted work. JPL reports research grants from Carl Zeiss Meditec; personal/consulting or advisory fees from Biotex Inc.; honoraria from TME Pharma, Carl Zeiss Meditec, and Siemens Healthineers; travel support from Biotex Inc., Carl Zeiss Meditec, and TME Pharma; and owns stock in Bayer, BioNTech, Siemens Healthineers, and TME Pharma outside the submitted work. The remaining authors report no conflict of interest related to this work.
