# Supplementary material for: Discordance between clinician- and patient-reported outcomes during radiotherapy for brain metastases: the prospective NeuroPRO study
Source: Clin Transl Radiat Oncol. 2026 Sep 8;62:101283. doi: 10.1016/j.ctro.2026.101283 (PMC13579688; doi:10.1016/j.ctro.2026.101283)
Supplement: Supplementary file 1 [file mmc1.docx]

***Supplement A***. Clinician-reported outcome questionnaire (NCI CTCAE v5.0).

***Supplement B***. Patient-reported outcome questionnaires (EORTC QLQ C30 and BN20, Ottawa Decision Regret Scale).

***Supplement C***. Matching of clinician-reported outcome (CRO) items with corresponding patient-reported outcome (PRO) questions assessing the same symptom or a closely corresponding functional domain. ADL, activities of daily living; n/a, not available.

| **PRO** | **Corresponding CRO** |
| --- | --- |
| 1. Do you have any trouble doing strenuous activities, like carrying a heavy shopping bag or a suitcase? | Fatigue |
| 2. Do you have any trouble taking a long walk? |  |
| 3. Do you have any trouble taking a short walk outside of the house? |  |
| 4. Do you need to stay in bed or a chair during the day? |  |
| 10. Did you need to rest? |  |
| 12. Have you felt weak? |  |
| 18. Were you tired? |  |
| 5. Do you need help with eating, dressing, washing yourself or using the toilet? | Limitations ADL |
| 6. Were you limited in doing either your work or other daily activities? |  |
| 7. Were you limited in pursuing your hobbies or other  leisure time activities? |  |
| 8. Have you had pain? | Dyspnea |
| 9. Have you had pain? | Pain |
| 19. Did pain interfere with your daily activities? |  |
| 11. Have you had trouble sleeping? | Insomnia |
| 13. Have you lacked appetite? | Anorexia, appetite loss |
| 14. Have you felt nauseated? | Nausea |
| 15. Have you vomited? | Emesis, vomiting |
| 16. Have you been constipated? | Obstipation, constipation |
| 17. Have you had diarrhea? | Diarrhea |
| 20. Have you had difficulty in concentrating on things, like reading a newspaper or watching television? | Concentration |
| 21. Did you feel tense? | Distress |
| 23. Did you feel depressed? |  |
| 22. Did you worry? | Worry |
| 24. Did you feel depressed? | Depression |
| 25. Have you had difficulty remembering things? | Memory |
| 26. Has your physical condition or medical treatment interfered with your family life? | n/a |
| 27. Has your physical condition or medical treatment interfered with your social activities? | n/a |
| 28. Has your physical condition or medical treatment caused you financial difficulties? | n/a |
| 29. How would you rate your overall health during the past week? | n/a |
| 30. How would you rate your overall quality of life during the past week? | n/a |
| 31. Did you feel uncertain about the future? | n/a |
| 32. Did you feel you had setbacks in your condition? | n/a |
| 33. Were you concerned about disruption of family life? | n/a |
| 34. Did you have headaches? | Headache |
| 35. Did your outlook on the future worsen? | n/a |
| 36. Did you have double vision? | Double vision |
| 37. Was your vision blurred? | Blurred vision, visual field problems |
| 38. Did you have difficulty reading because of your vision? |  |
| 39. Did you have seizures? | Seizures |
| 40. Did you have weakness on one side of your body? | Muscular weakness |
| 41. Did you have trouble finding the right words to  express yourself? | Difficulty finding words |
| 43. Did you have trouble communicating your thoughts? |  |
| 42. Did you have difficulty speaking? | Difficulty speaking |
| 44. Did you feel drowsy during the daytime? | Drowsiness |
| 45. Did you have trouble with your coordination? | Coordination problems |
| 46. Did hair loss bother you? | Hair loss |
| 47. Did itching of your skin bother you? | Itchy skin |
| 48. Did you have weakness of both legs? | Weakness in arms or legs |
| 49. Did you feel unsteady on your feet? |  |
| 50. Did you have trouble controlling your bladder? | Bladder control |
| 51. Did you have trouble hearing? | Hearing impairment, difficulty understanding speech |
| 52. Did you have numbness in your hands or feet? | Numbness, tingling in hands or feet |

***Supplement D***. Longitudinal assessment of discrepancies between CRO and PRO for 32 individual items. CRO, clinician-reported outcome; PRO, patient-reported outcome.

***Supplement E***. Chance-corrected agreement (Gwet’s AC2) between clinician-reported outcomes (CRO) and patient-reported outcomes (PRO) for 32 individual items, pooled across all timepoints. AC2 ranges from 0 (agreement no better than chance) to 1 (perfect agreement); agreement categories follow Landis & Koch benchmarks (poor ≤ 0, slight 0.01–0.20, fair 0.21–0.40, moderate 0.41–0.60, substantial 0.61–0.80, almost perfect 0.81–1.00) and are provided for descriptive orientation only. p-values test the null hypothesis that AC2 equals 0 (ie, agreement no better than chance), based on the standard error of the AC2 estimate. CI, confidence interval; underst., understanding; ADL, activities of daily living.

| **Symptom** | ***n*** | **AC2** | **95% CI** | ***p*** | **Agreement** |
| --- | --- | --- | --- | --- | --- |
| Fatigue | 132 | 0.73 | 0.66–0.79 | **< 0.001** | substantial |
| Depression | 129 | 0.39 | 0.23–0.56 | **< 0.001** | fair |
| Insomnia | 130 | 0.47 | 0.31–0.64 | **< 0.001** | moderate |
| Dyspnea | 132 | 0.50 | 0.35–0.65 | **< 0.001** | moderate |
| Appetite loss | 128 | 0.78 | 0.70–0.86 | **< 0.001** | substantial |
| Nausea | 130 | 0.92 | 0.87–0.96 | **< 0.001** | almost perfect |
| Vomiting | 127 | 0.96 | 0.93–0.99 | **< 0.001** | almost perfect |
| Constipation | 129 | 0.91 | 0.86–0.95 | **< 0.001** | almost perfect |
| Diarrhea | 129 | 0.86 | 0.80–0.92 | **< 0.001** | almost perfect |
| Concentration | 131 | 0.62 | 0.49–0.74 | **< 0.001** | substantial |
| Worry | 131 | 0.30 | 0.12–0.48 | **< 0.001** | fair |
| Memory | 130 | 0.67 | 0.57–0.76 | **< 0.001** | substantial |
| Headache | 121 | 0.88 | 0.82–0.94 | **< 0.001** | almost perfect |
| Double vision | 121 | 0.90 | 0.85–0.96 | **< 0.001** | almost perfect |
| Blurred vision | 124 | 0.81 | 0.73–0.90 | **< 0.001** | almost perfect |
| Visual field problems | 123 | 0.70 | 0.58–0.81 | **< 0.001** | substantial |
| Difficulty finding words | 125 | 0.77 | 0.69–0.86 | **< 0.001** | substantial |
| Difficulty speaking | 126 | 0.87 | 0.81–0.93 | **< 0.001** | almost perfect |
| Difficulty underst. speech | 124 | 0.79 | 0.71–0.88 | **< 0.001** | substantial |
| Drowsiness | 125 | 0.44 | 0.28–0.60 | **< 0.001** | moderate |
| Coordination problems | 123 | 0.68 | 0.57–0.80 | **< 0.001** | substantial |
| Distress | 133 | 0.47 | 0.33–0.62 | **< 0.001** | moderate |
| Bladder control | 123 | 0.87 | 0.81–0.94 | **< 0.001** | almost perfect |
| Hearing impairment | 125 | 0.74 | 0.64–0.84 | **< 0.001** | substantial |
| Numbness, tingling hands/feet | 126 | 0.73 | 0.62–0.83 | **< 0.001** | substantial |
| Weakness in legs or arms | 126 | 0.78 | 0.69–0.87 | **< 0.001** | substantial |
| Muscular weakness | 128 | 0.51 | 0.36–0.66 | **< 0.001** | moderate |
| Itchy skin | 121 | 0.89 | 0.84–0.93 | **< 0.001** | almost perfect |
| Hair loss | 124 | 0.76 | 0.66–0.86 | **< 0.001** | substantial |
| ADL | 131 | 0.71 | 0.64–0.78 | **< 0.001** | substantial |
| Seizures | 122 | 0.94 | 0.90–0.97 | **< 0.001** | almost perfect |
| Pain | 133 | 0.65 | 0.55–0.76 | **< 0.001** | substantial |

***Supplement F***. Chance-corrected agreement (Gwet’s AC2) between CRO and PRO for 32 individual items, stratified by timepoint. p-values test the null hypothesis that AC2 equals 0 (ie, agreement no better than chance), based on the standard error of the AC2 estimate. CRO, clinician-reported outcome; PRO, patient-reported outcome; CI, confidence interval; RT, radiotherapy; ADL, activities of daily living.

| **Symptom** | **Visit** | ***n*** | **AC2** | **95% CI** | ***p*** |
| --- | --- | --- | --- | --- | --- |
| Fatigue | Baseline | 60 | 0.70 | 0.59–0.81 | **< 0.001** |
|  | End of RT | 37 | 0.74 | 0.63–0.86 | **< 0.001** |
|  | Follow-up | 35 | 0.77 | 0.67–0.88 | **< 0.001** |
| Depression | Baseline | 59 | 0.39 | 0.15–0.64 | **0.001** |
|  | End of RT | 36 | 0.35 | -0.02–0.71 | **0.031** |
|  | Follow-up | 34 | 0.47 | 0.19–0.74 | **< 0.001** |
| Insomnia | Baseline | 60 | 0.44 | 0.18–0.69 | **< 0.001** |
|  | End of RT | 36 | 0.35 | -0.01–0.71 | **0.026** |
|  | Follow-up | 34 | 0.68 | 0.44–0.92 | **< 0.001** |
| Dyspnea | Baseline | 60 | 0.50 | 0.27–0.72 | **< 0.001** |
|  | End of RT | 37 | 0.41 | 0.07–0.74 | **0.009** |
|  | Follow-up | 35 | 0.60 | 0.35–0.85 | **< 0.001** |
| Appetite loss | Baseline | 60 | 0.83 | 0.74–0.93 | **< 0.001** |
|  | End of RT | 36 | 0.71 | 0.51–0.92 | **< 0.001** |
|  | Follow-up | 32 | 0.76 | 0.56–0.95 | **< 0.001** |
| Nausea | Baseline | 60 | 0.91 | 0.84–0.98 | **< 0.001** |
|  | End of RT | 36 | 0.90 | 0.80–1.00 | **< 0.001** |
|  | Follow-up | 34 | 0.94 | 0.89–0.99 | **< 0.001** |
| Vomiting | Baseline | 59 | 0.96 | 0.92–1.00 | **< 0.001** |
|  | End of RT | 36 | 0.95 | 0.88–1.00 | **< 0.001** |
|  | Follow-up | 32 | 0.95 | 0.89–1.00 | **< 0.001** |
| Constipation | Baseline | 60 | 0.76 | 0.62–0.89 | **< 0.001** |
|  | End of RT | 36 | 0.93 | 0.87–0.99 | **< 0.001** |
|  | Follow-up | 33 | 0.90 | 0.78–1.00 | **< 0.001** |
| Diarrhea | Baseline | 59 | 0.87 | 0.78–0.95 | **< 0.001** |
|  | End of RT | 36 | 0.80 | 0.63–0.96 | **< 0.001** |
|  | Follow-up | 34 | 0.91 | 0.84–0.98 | **< 0.001** |
| Concentration | Baseline | 60 | 0.61 | 0.43–0.79 | **< 0.001** |
|  | End of RT | 37 | 0.57 | 0.33–0.81 | **< 0.001** |
|  | Follow-up | 34 | 0.70 | 0.45–0.96 | **< 0.001** |
| Worry | Baseline | 60 | 0.38 | 0.12–0.65 | **0.003** |
|  | End of RT | 37 | 0.30 | -0.04–0.63 | **0.039** |
|  | Follow-up | 34 | 0.22 | -0.16–0.60 | 0.123 |
| Memory | Baseline | 59 | 0.52 | 0.33–0.71 | **< 0.001** |
|  | End of RT | 37 | 0.74 | 0.60–0.88 | **< 0.001** |
|  | Follow-up | 34 | 0.83 | 0.74–0.92 | **< 0.001** |
| Headache | Baseline | 55 | 0.86 | 0.75–0.97 | **< 0.001** |
|  | End of RT | 32 | 0.77 | 0.61–0.92 | **< 0.001** |
|  | Follow-up | 34 | 0.76 | 0.60–0.93 | **< 0.001** |
| Double vision | Baseline | 56 | 0.95 | 0.90–0.99 | **< 0.001** |
|  | End of RT | 33 | 0.82 | 0.66–0.98 | **< 0.001** |
|  | Follow-up | 32 | 0.92 | 0.81–1.00 | **< 0.001** |
| Blurred vision | Baseline | 55 | 0.86 | 0.75–0.96 | **< 0.001** |
|  | End of RT | 35 | 0.70 | 0.47–0.93 | **< 0.001** |
|  | Follow-up | 34 | 0.84 | 0.68–0.99 | **< 0.001** |
| Visual field problems | Baseline | 57 | 0.76 | 0.61–0.90 | **< 0.001** |
|  | End of RT | 33 | 0.45 | 0.13–0.77 | **0.004** |
|  | Follow-up | 33 | 0.82 | 0.66–0.98 | **< 0.001** |
| Difficulty finding words | Baseline | 57 | 0.82 | 0.72–0.93 | **< 0.001** |
|  | End of RT | 34 | 0.69 | 0.49–0.90 | **< 0.001** |
|  | Follow-up | 34 | 0.76 | 0.60–0.93 | **< 0.001** |
| Difficulty speaking | Baseline | 57 | 0.88 | 0.80–0.96 | **< 0.001** |
|  | End of RT | 35 | 0.84 | 0.70–0.97 | **< 0.001** |
|  | Follow-up | 34 | 0.87 | 0.74–0.99 | **< 0.001** |
| Difficulty understanding speech | Baseline | 57 | 0.77 | 0.64–0.91 | **< 0.001** |
|  | End of RT | 34 | 0.74 | 0.58–0.91 | **< 0.001** |
|  | Follow-up | 33 | 0.87 | 0.75–0.99 | **< 0.001** |
| Drowsiness | Baseline | 56 | 0.42 | 0.15–0.69 | **0.001** |
|  | End of RT | 35 | 0.53 | 0.28–0.77 | **< 0.001** |
|  | Follow-up | 34 | 0.47 | 0.21–0.73 | **< 0.001** |
| Coordination problems | Baseline | 55 | 0.75 | 0.59–0.90 | **< 0.001** |
|  | End of RT | 34 | 0.52 | 0.25–0.80 | **< 0.001** |
|  | Follow-up | 34 | 0.71 | 0.48–0.94 | **< 0.001** |
| Distress | Baseline | 61 | 0.51 | 0.30–0.71 | **< 0.001** |
|  | End of RT | 37 | 0.50 | 0.25–0.74 | **< 0.001** |
|  | Follow-up | 35 | 0.43 | 0.10–0.76 | **0.007** |
| Bladder control | Baseline | 57 | 0.86 | 0.77–0.96 | **< 0.001** |
|  | End of RT | 32 | 0.88 | 0.76–1.00 | **< 0.001** |
|  | Follow-up | 34 | 0.89 | 0.78–1.00 | **< 0.001** |
| Hearing impairment | Baseline | 58 | 0.76 | 0.61–0.90 | **< 0.001** |
|  | End of RT | 34 | 0.75 | 0.55–0.94 | **< 0.001** |
|  | Follow-up | 33 | 0.70 | 0.47–0.93 | **< 0.001** |
| Numbness, tingling hands/feet | Baseline | 57 | 0.71 | 0.55–0.88 | **< 0.001** |
|  | End of RT | 35 | 0.65 | 0.40–0.90 | **< 0.001** |
|  | Follow-up | 34 | 0.83 | 0.69–0.97 | **< 0.001** |
| Weakness in legs or arms | Baseline | 57 | 0.82 | 0.71–0.93 | **< 0.001** |
|  | End of RT | 35 | 0.67 | 0.40–0.94 | **< 0.001** |
|  | Follow-up | 34 | 0.80 | 0.64–0.96 | **< 0.001** |
| Muscular weakness | Baseline | 58 | 0.54 | 0.33–0.75 | **< 0.001** |
|  | End of RT | 35 | 0.42 | 0.15–0.69 | **0.002** |
|  | Follow-up | 35 | 0.59 | 0.31–0.86 | **< 0.001** |
| Itchy skin | Baseline | 54 | 0.74 | 0.59–0.89 | **< 0.001** |
|  | End of RT | 33 | 0.88 | 0.76–0.99 | **< 0.001** |
|  | Follow-up | 34 | 0.74 | 0.57–0.92 | **< 0.001** |
| Hair loss | Baseline | 56 | 0.79 | 0.66–0.92 | **< 0.001** |
|  | End of RT | 34 | 0.77 | 0.56–0.98 | **< 0.001** |
|  | Follow-up | 34 | 0.70 | 0.49–0.92 | **< 0.001** |
| ADL | Baseline | 60 | 0.72 | 0.61–0.83 | **< 0.001** |
|  | End of RT | 37 | 0.72 | 0.58–0.85 | **< 0.001** |
|  | Follow-up | 34 | 0.71 | 0.58–0.83 | **< 0.001** |
| Seizures | Baseline | 56 | 0.91 | 0.83–0.99 | **< 0.001** |
|  | End of RT | 33 | 0.94 | 0.86–1.00 | **< 0.001** |
|  | Follow-up | 33 | 0.94 | 0.88–1.00 | **< 0.001** |
| Pain | Baseline | 60 | 0.60 | 0.43–0.78 | **< 0.001** |
|  | End of RT | 37 | 0.69 | 0.50–0.88 | **< 0.001** |
|  | Follow-up | 36 | 0.70 | 0.54–0.86 | **< 0.001** |

***Supplement G***. Range of chance-corrected agreement (Gwet’s AC2) between CRO and PRO across the three assessment timepoints (baseline, end of treatment, follow-up) for 32 individual items. Minimum and maximum AC2 denote the lowest and highest of the three timepoint-specific AC2 values per item; spread (Δ AC2) is their difference and quantifies the temporal variability of agreement, with items sorted in descending order of spread. CRO, clinician-reported outcome; PRO, patient-reported outcome; ADL, activities of daily living.

| **Symptom** | **Minimum AC2** | **Maximum AC2** | **Spread (Δ AC2)** |
| --- | --- | --- | --- |
| Visual field problems | 0.45 | 0.82 | 0.37 |
| Insomnia | 0.35 | 0.68 | 0.33 |
| Memory | 0.52 | 0.83 | 0.30 |
| Coordination problems | 0.52 | 0.75 | 0.22 |
| Dyspnea | 0.41 | 0.60 | 0.20 |
| Numbness, tingling in hands or feet | 0.65 | 0.83 | 0.18 |
| Constipation | 0.76 | 0.93 | 0.18 |
| Muscular weakness | 0.42 | 0.58 | 0.17 |
| Worry | 0.22 | 0.38 | 0.16 |
| Blurred vision | 0.70 | 0.86 | 0.16 |
| Weakness in legs or arms | 0.67 | 0.82 | 0.15 |
| Itchy skin | 0.74 | 0.88 | 0.14 |
| Difficulty finding words | 0.69 | 0.82 | 0.13 |
| Concentration | 0.57 | 0.70 | 0.13 |
| Double vision | 0.82 | 0.95 | 0.13 |
| Difficulty understanding speech | 0.74 | 0.87 | 0.13 |
| Appetite loss | 0.71 | 0.83 | 0.12 |
| Depression | 0.35 | 0.47 | 0.12 |
| Diarrhea | 0.80 | 0.91 | 0.12 |
| Drowsiness | 0.42 | 0.53 | 0.10 |
| Headache | 0.76 | 0.86 | 0.10 |
| Pain | 0.60 | 0.70 | 0.10 |
| Hair loss | 0.70 | 0.79 | 0.08 |
| Distress | 0.43 | 0.51 | 0.08 |
| Fatigue | 0.70 | 0.78 | 0.07 |
| Hearing impairment | 0.70 | 0.76 | 0.06 |
| Difficulty speaking | 0.83 | 0.88 | 0.05 |
| Nausea | 0.90 | 0.94 | 0.04 |
| Seizures | 0.91 | 0.94 | 0.04 |
| Bladder control | 0.86 | 0.89 | 0.03 |
| Vomiting | 0.95 | 0.96 | 0.01 |
| ADL | 0.71 | 0.72 | 0.01 |

***Supplement H***. Agreement between CRO and PRO for single items across different timepoints. CRO, clinician-reported outcome; PRO, patient-reported outcome; CI, confidence interval; LoA, limit of agreement; FU, follow-up; s, significant (*p* < 0.05); ns, not significant (*p* ≥ 0.05).

| **Symptom** | **Interaction *p*** | **Timepoint *p*** | **Group *p*** | **Mean**  **CRO-PRO**  **difference** | **95% CI lower** | **95% CI upper** | **Bland-Altman bias** | **LoA lower** | **LoA upper** | **Tukey *p* (pre/post/FU)** |
| --- | --- | --- | --- | --- | --- | --- | --- | --- | --- | --- |
| Fatigue | 0.410 | **< 0.001** | 0.187 | 0.13 | –0.07 | 0.34 | 0.13 | –0.20 | 0.47 | ns/ns/ns |
| Depression | 0.425 | 0.076 | **< 0.001** | 0.75 | 0.54 | 0.97 | 0.75 | 0.39 | 1.12 | **s**/**s**/**s** |
| Insomnia | 0.197 | **0.029** | **< 0.001** | 0.76 | 0.53 | 0.98 | 0.76 | 0.22 | 1.29 | **s**/**s**/**s** |
| Dyspnea | 0.857 | 0.458 | **< 0.001** | **1.00** | 0.78 | 1.21 | **1.00** | 0.85 | 1.14 | **s**/**s**/**s** |
| Appetite loss | 0.852 | 0.324 | **< 0.001** | 0.59 | 0.41 | 0.76 | 0.59 | 0.47 | 0.70 | **s**/**s**/**s** |
| Nausea | 0.958 | 0.432 | **0.001** | 0.29 | 0.11 | 0.46 | 0.29 | 0.23 | 0.34 | **s**/ns/ns |
| Vomiting | 0.947 | 0.252 | **0.009** | 0.18 | 0.05 | 0.32 | 0.18 | 0.13 | 0.24 | ns/ns/ns |
| Constipation | 0.895 | 0.373 | **< 0.001** | 0.41 | 0.28 | 0.55 | 0.41 | 0.34 | 0.48 | **s**/**s**/**s** |
| Diarrhea | 0.597 | 0.846 | **< 0.001** | 0.41 | 0.24 | 0.57 | 0.41 | 0.19 | 0.62 | **s**/**s**/ns |
| Concentration | 0.213 | **0.048** | 0.939 | 0.23 | 0.01 | 0.45 | 0.23 | –0.26 | 0.72 | ns/ns/**s** |
| Worry | 0.596 | 0.063 | **< 0.001** | 0.64 | 0.41 | 0.89 | 0.64 | 0.27 | 1.01 | **s**/**s**/**s** |
| Memory | 0.822 | 0.208 | **0.021** | 0.26 | 0.04 | 0.48 | 0.26 | 0.08 | 0.44 | ns/ns/ns |
| Headache | 0.840 | 0.669 | **0.002** | 0.26 | 0.09 | 0.42 | 0.26 | 0.14 | 0.37 | ns/**s**/ns |
| Double vision | 0.586 | 0.645 | **0.011** | 0.23 | 0.05 | 0.42 | 0.23 | 0.02 | 0.45 | ns/**s**/ns |
| Blurred vision | 0.428 | 0.571 | **0.002** | 0.34 | 0.13 | 0.54 | 0.34 | –0.01 | 0.68 | ns/**s**/ns |
| Visual field problems | 0.351 | 0.471 | **< 0.001** | 0.60 | 0.38 | 0.81 | 0.60 | 0.20 | 0.99 | **s**/**s**/**s** |
| Difficulty finding words | 0.292 | 0.426 | **< 0.001** | 0.71 | 0.56 | 0.87 | 0.71 | 0.43 | 1.00 | **s**/**s**/**s** |
| Difficulty speaking | 0.789 | 0.645 | **< 0.001** | 0.37 | 0.21 | 0.53 | 0.37 | 0.24 | 0.50 | **s**/**s**/**s** |
| Difficulty understanding speech | 0.270 | 0.671 | **< 0.001** | 0.62 | 0.47 | 0.78 | 0.62 | 0.28 | 0.97 | **s**/**s**/**s** |
| Drowsiness | 0.590 | **0.006** | **< 0.001** | 0.75 | 0.54 | 0.96 | 0.75 | 0.48 | 1.02 | **s**/**s**/**s** |
| Coordination problems | 0.450 | 0.075 | **< 0.001** | 0.70 | 0.50 | 0.91 | 0.70 | 0.41 | 1.00 | **s**/**s**/**s** |
| Distress | 0.170 | 0.082 | **< 0.001** | 0.37 | 0.16 | 0.58 | 0.37 | –0.11 | 0.85 | ns/ns/**s** |
| Bladder control | 0.894 | 0.719 | **< 0.001** | 0.44 | 0.29 | 0.58 | 0.44 | 0.35 | 0.53 | **s**/**s**/**s** |
| Hearing impairment | 0.791 | 0.747 | **< 0.001** | 0.63 | 0.43 | 0.82 | 0.63 | 0.45 | 0.80 | **s**/**s**/**s** |
| Numbness, tingling in hands or feet | 0.580 | 0.615 | **< 0.001** | 0.66 | 0.47 | 0.85 | 0.66 | 0.40 | 0.92 | **s**/**s**/**s** |
| Weakness in legs or arms | 0.952 | **0.044** | 0.386 | 0.10 | –0.12 | 0.32 | 0.10 | 0.02 | 0.17 | ns/ns/ns |
| Muscular weakness | 0.967 | **0.015** | **< 0.001** | 0.65 | 0.41 | 0.88 | 0.65 | 0.58 | 0.71 | **s**/**s**/**s** |
| Itchy skin | 0.969 | 0.853 | **< 0.001** | 0.42 | 0.29 | 0.55 | 0.42 | 0.38 | 0.46 | **s**/**s**/**s** |
| Hair loss | 0.523 | **0.016** | **0.035** | 0.22 | 0.02 | 0.42 | 0.22 | –0.04 | 0.48 | **s**/ns/ns |
| ADL | 0.694 | **0.004** | 0.833 | 0.02 | –0.19 | 0.24 | 0.02 | –0.22 | 0.26 | ns/ns/ns |
| Seizures | 0.906 | 0.481 | 0.193 | 0.09 | –0.05 | 0.23 | 0.09 | 0.02 | 0.16 | ns/ns/ns |
| Pain | 0.932 | 0.590 | **< 0.001** | 0.50 | 0.31 | 0.68 | 0.50 | 0.41 | 0.58 | **s**/**s**/**s** |

**References:**

- National Cancer Institute (2017). Common Terminology Criteria for Adverse Events (CTCAE), version 5.0. U.S. Department of Health and Human Services.

- Aaronson, N.K., et al. (1993). The European Organization for Research and Treatment of Cancer QLQ-C30: a quality-of-life instrument for use in international clinical trials in oncology. J Natl Cancer Inst. 85(5):365–376.

- Osoba, D., et al. (1996). The development and psychometric validation of a brain cancer quality-of-life questionnaire for use in combination with general cancer-specific questionnaires. Qual of Life Res. 5: 139–150.

- Brehaut, J.C., et al. (2003). Validation of a decision regret scale. Med Decis Making. 23(4):281–292.
